# A Solvent-Free Surface Suspension Melt Technique for Making Biodegradable PCL Membrane Scaffolds for Tissue Engineering Applications

**DOI:** 10.3390/molecules21030386

**Published:** 2016-03-21

**Authors:** Ratima Suntornnond, Jia An, Ajay Tijore, Kah Fai Leong, Chee Kai Chua, Lay Poh Tan

**Affiliations:** 1 Singapore Centre for 3D Printing, School of Mechanical and Aerospace Engineering, Nanyang Technological University, Block N3.1, 50 Nanyang Avenue, Singapore 639798, Singapore; ANJIA@ntu.edu.sg (A.J.); MKFLEONG@ntu.edu.sg (K.F.L.); MCKCHUA@ntu.edu.sg (C.K.C.); 2 School of Materials Science and Engineering, Nanyang Technological University, Block N4.1, 50 Nanyang Avenue, Singapore 639798, Singapore; AJAY0009@e.ntu.edu.sg (A.T.); LPTAN@ntu.edu.sg (L.P.T.)

**Keywords:** biodegradable polymers, polycaprolactone, polymer membranes, tissue engineering

## Abstract

In tissue engineering, there is limited availability of a simple, fast and solvent-free process for fabricating micro-porous thin membrane scaffolds. This paper presents the first report of a novel surface suspension melt technique to fabricate a micro-porous thin membrane scaffolds without using any organic solvent. Briefly, a layer of polycaprolactone (PCL) particles is directly spread on top of water in the form of a suspension. After that, with the use of heat, the powder layer is transformed into a melted layer, and following cooling, a thin membrane is obtained. Two different sizes of PCL powder particles (100 µm and 500 µm) are used. Results show that membranes made from 100 µm powders have lower thickness, smaller pore size, smoother surface, higher value of stiffness but lower ultimate tensile load compared to membranes made from 500 µm powder. C2C12 cell culture results indicate that the membrane supports cell growth and differentiation. Thus, this novel membrane generation method holds great promise for tissue engineering.

## 1. Introduction

Biodegradable porous membranes have been used for many applications, especially for tissue engineering (TE) and biomedical applications [1]. Over the past decades, there have been many reports that have shown potential of biodegradable membrane as TE scaffolds in different types of organs such as blood vessels [2], heart tissue [3] and muscle [4]. The methods that are currently available to fabricate membranes for TE application are summarized in Table 1.

The most common method is solution casting or solvent casting [5,6], in which a volatile organic solvent is used to dissolve the solid polymer material. Then, the polymer mixture is poured into a glass container and after the solvent fully evaporates, a solid film is formed. Solution casting is simple and versatile, but the drawback is that solution-casted films usually require certain period of time for post-processing, for example, they typically need 1–2 days to get rid of all traces of solvent and a limited number of pores are created [7]. Although a solvent cast film can have patterned pore structures when prepared by modified methods such as the selective wetted surface method [8] or colloidal systems [9], an organic solvent is still required.

Biaxial drawing is a method using mechanical forces to stretch a nonporous film in perpendicular directions so that an ultrathin film can be formed by enlarging the area of the film [5,14]. Films that have been prepared using a two-roll milling method do not require the use of organic solvents, however, they need to undergo biaxial stretching to generate ultra-thin films [5]. These also do not have pores to allow cell interactions among layers. For both the solvent casting and biaxial stretching method, further steps have to be taken in order to create a porous structure on the surface. Usually, solvent casting and biaxial method films can be post-processed by laser surface modification [15] or a robotic perforation system [10]. Electrospinning methods have also shown their potential for tissue engineering applications [16]. Electrospun scaffolds consist of randomly stacked microfibers. The fibers in electrospun scaffolds are very thin, which can yield micro to nano-fibers. [12,13,17]. Like solution casting, electrospinning processes usually require the use of an organic solvent to dissolve a biodegradable polymer such as polycaprolactone, PCL [17,18] or poly(L-lactic) acid, PLLA [19]. In order to use these membranes for TE scaffolds, a post-processing step for solvent removal is necessary to ensure the cell compatibility of the membrane. In summary, only a few membrane fabrication methods that do not need further processing to create pores or solvent removal have been reported.

In this paper, we demonstrate a novel method for fabricating a solvent-free, micro-porous, biodegradable membrane by a surface suspension melt technique. This method eliminates the use of organic solvent by using water instead. The water acts as medium and platform for particles so this mixture becomes a suspension system. Based on particle size there are three types of mixtures, including solutions, colloids and suspensions. Suspensions normally refer to mixtures of liquids and coarse solid particles (size > 1 micrometer) which particles can be seen on a macroscopic scale. Eventually after some period of time suspension particles will sink. However, in this experiment, the particles stay only on the top of the water surface so the mixture becomes a “surface suspension system”. Moreover, we also report possible applications of our solvent-free micro-porous membrane as a tissue engineering scaffold. By seeding C2C12 mouse myoblasts which are a potential muscle cell line that can be expanded and isolated *in vitro* [20,21], cell proliferation and differentiation on the membrane surface were investigated.

## 2. Results and Discussion

### 2.1. Formation of a Layer of PCL Particles

**Figure 1:**
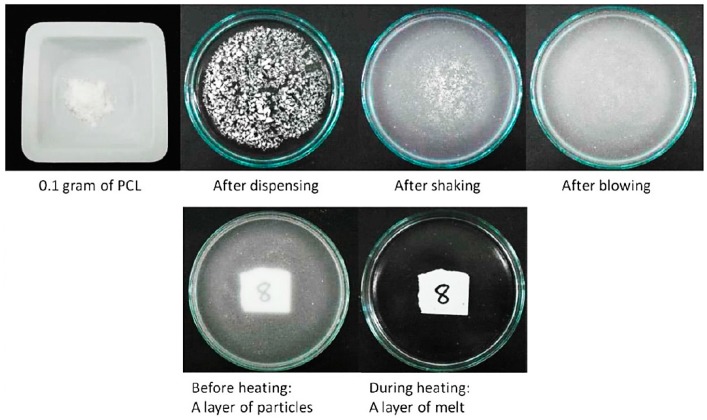
Preparation of a layer of PCL particles on the water surface and melting state layer formation in a 10 cm diameter glass dish.

In this paper, a novel method of fabricating PCL micro-porous membranes is reported. It involves three steps: preparation of a layer of particles on a water surface to form a suspension mixture, heating this to form a melted layer, and cooling to form a membrane layer. Figure 1 shows a layer of PCL particles during preparation. Dispensing was completed by gently tapping the weighing boat. The glass dish was gently shaken with repeated circular motions. Distribution was aided by blowing with a repeatedly squeezed rubber bulb. Normally, suspension mixtures lead to the sedimentation of particles at the bottom of the glass Petri dish. However, PCL powder particles are small, light and highly hydrophobic, so 0.1 g of 100 µm PCL powder can float on the water surface because of the surface tension. The powders used in this experiment are not enough to agglomerate and sink. In the case of 500 µm powders, they behaved the same way as 100 µm particle size ones but required more powder (in terms of weight) to cover the same area.

### 2.2. Fabrication of a Membrane from a Layer of PCL Particles

**Figure 2:**
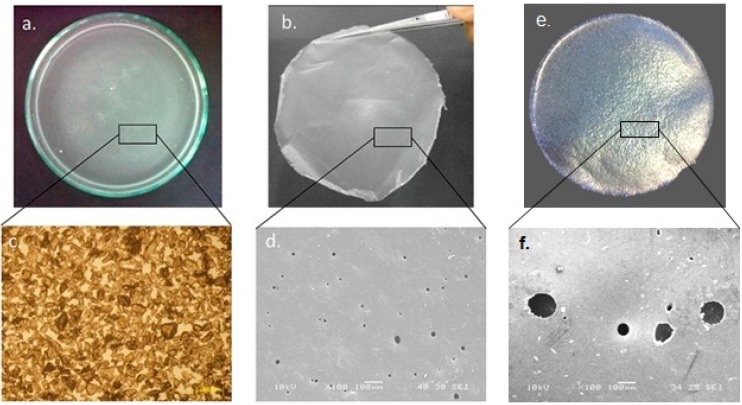
(**a**) Macroscopic of the powder dispersion on the water surface; (**b**) Macroscopic view of membrane made from 100 µm powder; (**c**) Microscopic image of the powder dispersion under a light microscope with ×10 magnification; (**d**) Microscopic image of membrane made from 100 µm powder surface (SEM at ×100); (**e**) Macroscopic image of membrane made from 500 µm powder; and (**f**) Microscopic image of membrane made from 500 µm powder surface (SEM at ×100).

The concept that a layer of micro-particles can be directly formed into a layer of membrane is demonstrated in Figure 1 and Figure 7. Both figures indicate that the membrane can be formed by the powder melting on top of water surface with the use of heat. In this method, water acts as a heat medium and as a platform for PCL particles in both the solid and liquid state. Water allows the particles to stay on top by the effect of surface tension between the hydrophobic polymer and water. Figure 2a shows a layer of dispensed particles spread on the water surface in the form of a surface suspension mixture. Figure 2c is a zoomed-in view that shows a microscopic observation of a layer of particles resting only on the water surface. The fabricated PCL membrane can be found in Figure 2b. It was semi-transparent and soft, and had a similar size as the glass dish. The zoom-in view shows the membrane is solid and micro-porous, as indicated by the microscopic view in Figure 2d.

During the fabrication process, a part of the powder used had not gone through to form the membrane but was blown out of the glass dish or stuck on the glass wall. The temperature of 80 °C was chosen due to the fact the melting point of polycaprolactone is around 60 °C and the boiling point of water is 100 °C. Figure 2 demonstrates that when the PCL powder layer was completely melted, it changed from an opaque solid to a transparent liquid. The fabrication temperature must be able to melt PCL particles whilst not boiling water. The fabrication time of 20 min was chosen as only a ring of particles near the wall of the glass dish melted and the entire layer of particles remained white and opaque at 10 min. At 30 min, the membrane was found to have shrunk in size due to melt surface tension. PCL is an adhesive material. When PCL is melted, it is able to adhere to glass. If the edge of the melted layer firmly attaches to the glass wall, the melted layer will not shrink as the attachment resists the contraction. However, if some part of the edge happens to detach from the glass wall due to insufficient coverage of powder and the resulting poor attachment, a hole may form near the edge of membrane. A smaller but thicker membrane can be formed after cooling as a result of the detachment. To prevent these problems, the blowing step in the preparation of a layer of particles is critical. Lastly, the glass border of the Petri dish not only limits the spread of the powder but also facilitates the circumferential attachment of a layer melt. These methods actually can be repeated and used for different size of particles. However, this method depends a lot on user experience and the way powders were dispensed on top of water still need further development for better precision.

### 2.3. Membrane Surface Characteristics and Mechanical Strength

The membrane characteristics and mechanical properties, including thickness, roughness, stiffness and ultimate tensile load are presented in Table 2.

**Figure 3:**
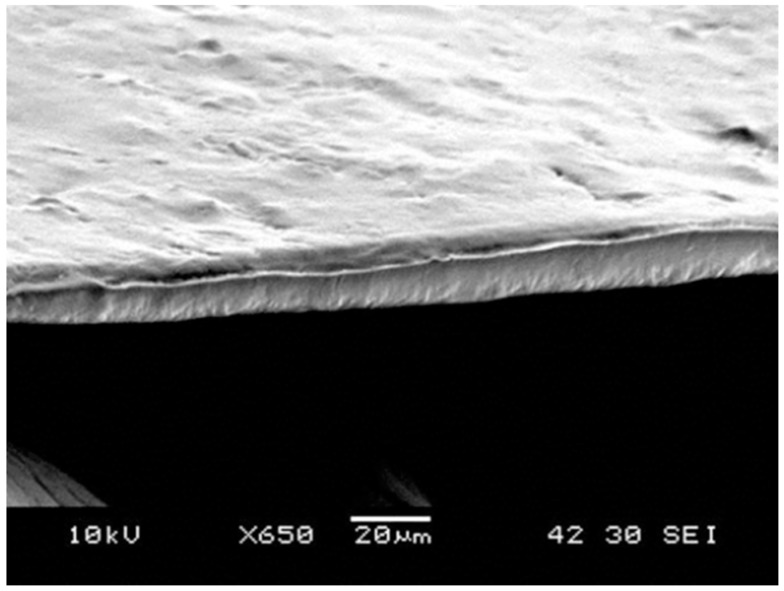
The cross-section of a PCL membrane made from 100 µm powder.

The membrane morphology in Figure 2 showed a comparison between the powder layer and the membrane layer. The initial powder layer consists of irregularly shaped particles and the membrane is a solid piece with pores. As the result of the membrane thickness presented in Table 2, the thickness value is much smaller than the size of the PCL particles used. Initially, particles should form a layer of a thickness of about 100 µm and 500 µm as well as a network of spaces among these particles due to their irregular shapes. When particles are melted on the water surface, they become round and spread, and fill in their adjacent spaces. The overall thickness of the layer is reduced as a result of powder redistribution. Moreover, Table 2 also shows that a bigger particle size leads to a thicker membrane. For mechanical properties, the thicker membrane resulted in a higher ultimate load, but lower stiffness. Figure 3 is a SEM image of the cross-section of a membrane, which shows the measured results. The measurement results of the membrane roughness may be used to explain the percepton of a fine texture on the membrane. The small size particles used in the fabrication resulted in an even-textured membrane surface. Moreover, the consistency in particle sizes and natural flatness of the water surface may also contribute to the overall evenness.

**Figure 4:**
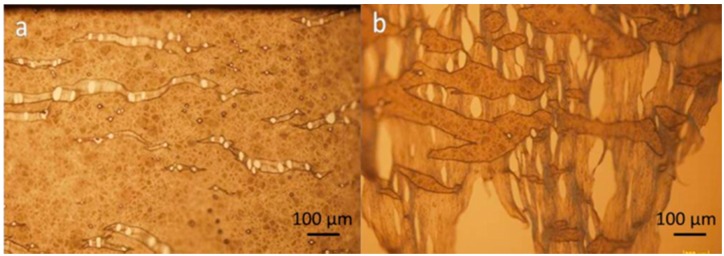
Failure mode (**a**) Near the sample holding grip (**b**) In the middle of a sample.

Figure 4 shows the structure of a stretched membrane. It can be seen that the pores were the place that cracks started to occur. Small cracks adjacent to each other converge into a long crack perpendicular to the loading. The thinning process usually started at the periphery of pores and regions with less or no pores decreased in size and became connected by mutiple thinned strips as shown in Figure 4b. This morphological observation indicates that when applying the membrane to a repair site, the wrapping process needs to be carefully handled with gentle stretching so that the enlargement of the pores on the membrane can be minimized.

### 2.4. Distribution of Pore Size

**Figure 5:**
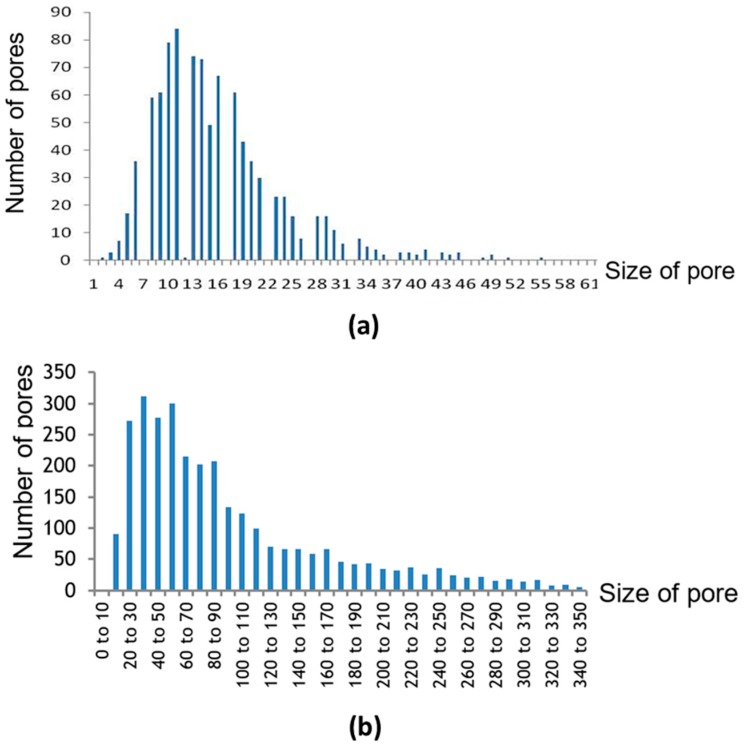
Pore size distribution of (**a**) membranes made from 100 µm powder (**b**) membranes made from 500 µm powder.

As shown in Figure 2d,f, black spots ing the membrane pores show that the pores’ size varied. Figure 5 and Table 3 summarise the pore size distribution of the three membranes. The pore size ranged from a few micrometers to nearly one hundred micrometers, and the average pore sizes for the three membranes were 16.4 ± 8.8 µm for membrane made from 100 µm powder and 95.4 ± 42.5 µm for membrane made from 500 µm powder, respectively. By comparing the pore size from membrane made from 100 µm and 500 µm powder in Table 3, the results show that a larger particle size leads to more variation in pore size. Smaller powder leads to more consistancy in membrane pore size. However, the pore distribution trend was the same for the two different membranes as illustrated in Figure 5.

### 2.5. In Vitro Cell Compatibility Study

**Figure 6:**
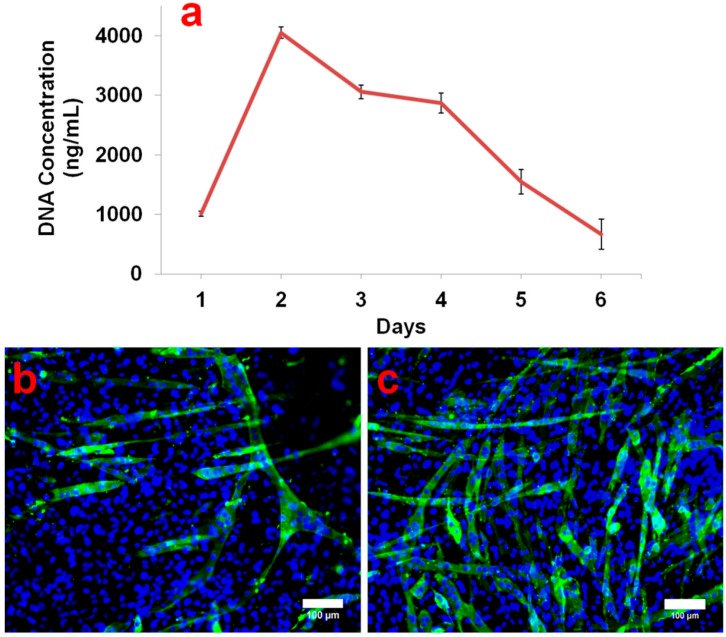
(**a**) DNA concentration of C2C12 myoblasts found at different time intervals in PicoGreen assay; (**b**) MHC staining (green) displayed myotube formation on PCL membrane after 4 days of cell seeding; (**c**) Positive control demonstrates induction of myotube formation in C2C12 myoblasts grown in DMEM containing 2% horse serum.

In order to investigate the cell proliferation rate, C2C12 myoblasts grown on biocompatible PCL membranes were subjected to a PicoGreen DNA quantification assay. Figure 6a shows the cell DNA concentration per PCL membrane at different time intervals. During the DNA quantification assay, the DNA concentration was found to be around 1000 ng/mL, 1 day after cell seeding. Remarkably, on day 2, there was a four-fold increase in the cell DNA concentration (4000 ng/mL). It has already been reported that the C2C12 doubling time is approximately 12 h [22,23]. Hence, the observed four-fold increase in DNA concentration after 24 h is feasible. On day 3, a sudden decline in DNA concentration was observed. Moreover, the DNA concentration gradually and continuously decreased till day 6 and finally it fell below 1000 ng/mL. The cell proliferation results showed that on day 2, C2C12 reached 100% confluence. This contact inhibition of C2C12 growth coupled with withdrawal from the proliferation cycle as myoblasts fused to form myotubes, causing a down-turn in cell population as seen from day 3 onwards. Nevertheless, the results from the first two days imply that the PCL membrane provides good support for cell attachment and proliferation.

MHC immunostaining of cells grown over PCL membranes after 4 days of culture showed distinct myotube formation (Figure 6b). Previous reports have elucidated the systematized skeletal myogenesis process and revealed the connection between the different steps that control the fusion of mononucleated myoblasts into terminally differentiated multinucleated myotubes [24,25]. Differentiated myotube formation over PCL membrane specifically shows the biocompatibility of PCL membrane for muscle tissue engineering. The positive control (Figure 6c) shows the myotube formation in C2C12 cells induced by 2% horse serum. These cells grew in the tissue culture plastic dish for 4 days before performing the immunostaining. Finally, the results of *in vitro* of C2C12 myoblast cell culture indicated that the membrane is able to support cell growth and differentiation.

The solvent-free surface suspension melt technique has a number of advantages. Firstly, the process does not involve any organic solvent or additives, which is particularly important for tissue engineering, because toxic chemical residues are likely to alter the behaviors of cells or cause cell death. In order to ensure that there is no organic solvent residue left, post-processing is required to remove all the residual solvent out of membrane. These post-processing steps can take around 1–2 days [7] so by using this method, post-processing is negligible. Secondly, it is fast. The method only requires a common laboratory oven and the membrane can be formed within less than 30 min. Thirdly, this method effectively utilizes the spaces among micro-particles, which eventually leads to the pores on the membrane, enabling the fabrication of a micro-porous membrane without the need for surface modification post-processing. Even though mechanical drilling and laser drilling can be used to confirm the uniformity of pore size and pore position on films or membranes, mechanical drilling can lead to problems of tool breakage and force-induced cutting. On the other hand, for laser drilling, many parameters have to be controlled carefully to avoid an issue of heat-affected zone (HAZ) that may affect materials’ properties [26,27]. Finally, the substrate in this method is a liquid. The fluidity of liquids allows convenient detachment of a flat membrane, avoiding bent or ruffled shapes. Therefore, this method can overcome the challenge of biodegradable membrane fabrication purifying post-processing because it can generate micro-porous membranes in a fast and straightforward manner without any use of organic solvent. However, some limitations should also be addressed. The materials have to be in the form of a fine powder, as coarse particles affect the texture of the membrane. Additionally, the powder has to be insoluble in the liquid substrate. Otherwise, the particles will dissolve in the liquid. Furthermore, the melting point of the powder has to be lower than the boiling point of the liquid substrate. Moreover, even though PCL has good biocompatibility, it also has poor strength after degradation. To overcome this problem, PCL can be blended with minerals or nanomaterials to enhance its mechanical properties. [28,29]. Lastly, the way powders were deposited onto water still needs improvement for better precision and uniformity. Future work will be focused on fabricating more precise pore structures by integrating this method with other techniques, for example, reverse engineering from CT-scan [30], bioprinting [31,32], solid-scaffold based additive manufacturing (AM) [33,34,35,36] and 4D printing [37].

## 3. Experimental Section

### 3.1. Polycaprolactone Membrane Fabrication

Polycaprolactone powder (CAPA^®^ 6501, Molecular weight: 50 kDa, average particle size 100 µm) was purchased from Solvay Interox, Warrington, UK. The material density of polycaprolactone is 1.1 g/cm^3^. The melting point is around 60°C. Another size of PCL powder is 500 µm PCL powder which was purchased from Perstorp (Malmö, Sweden).

**Figure 7:**
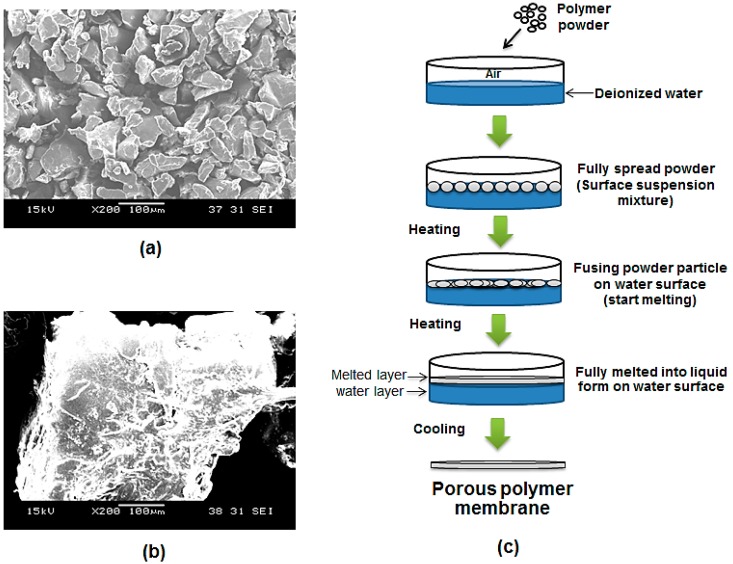
(**a**) SEM image of 100 µm dry PCL powder at ×200; (**b**) SEM image of 500 µm dry PCL powder at ×200; (**c**) Schematic illustration of a micro-porous membrane fabrication starting from a micro-particles powder layer forming a surface suspension mixture.

The CAPA^®^ PCL powder was sieved with a no. 140 sieve to confirm its 100 µm powder size. For 500 µm powders, the particle size can be confirmed by sieving with a no. 35 sieve as shown in the ×200 magnification SEM image in Figure 7a,b. As shown in Figure 7c, to facilitate the preparation of a layer of micro-particles, a liquid substrate was used as a platform for the powder and to allow an even distribution of powder particles as reported in our previous studies [38,39]. Briefly, 30 mL of deionized water was prepared and poured into a round glass dish (diameter: 10 cm) at room temperature. 100 µm PCL powder (0.1 g) and 500 µm PCL powder (0.7 g) was weighed and slowly dispensed onto the water surface to form a surface suspension system. The glass dish was then gently shaken circularly to allow the microparticles to distribute evenly on the water surface. A rubber bulb was used to gently blow off and spread the excess powder.

Subsequently, the glass dish was lightly shaken for another 10 s to allow the particles to distribute evenly. The next step for 100 µm powder, it was to place the glass dish on the bottom level of a laboratory oven (BINDER, Inc., Bohemia, NY, USA) and the temperature was set at 80 °C. After 20 min, the glass dish was taken out and cooled at room temperature until a solid white membrane is formed. On the other hand, for 500 µm powder, the glass dish which had powder suspend on top was put onto a laboratory heater (Thermolyne Cimarec^®^ 1, Thermo Scientific, Waltham, MA, USA) and the heater level was set at level 2 through all the experiment. After 15 min, the heater was turn off and the glass dish was removed from the heater. Finally, the glass dish was cooled down at room temperature. After about 5 min, a solid white membrane appeared on top of the water surface. In total, 20 membranes (ten membranes for each particle size) were fabricated continuously for all characterization tests.

### 3.2. Membrane Characterization

#### 3.2.1. Membrane Morphology

Light microscopy (CKX41, Olympus, Tokyo, Japan) was used to examine the morphology of particles on the water surface and the failure morphology was examined by scanning electron microscopy (SEM, JSM-5600LV, Jeol, Tokyo, Japan). The PCL membrane was cut into 10 mm × 10 mm square pieces and coated with gold at 10 mA for 20 s before the SEM examination.

#### 3.2.2. Thickness Measurement

A micrometer (Coolant Proof Micrometer Series 293, accuracy: ±0.00127 mm, Mitutoyo, Tokyo, Japan) was used to measure the thickness of six PCL membranes for each powder particle size. For each membrane, ten measurements were taken at points selected randomly.

#### 3.2.3. Pore Size Measurement

Three membranes for each size of powder particle were used in this characterization test. Each membrane was cut into five rectangular samples (25 mm × 15 mm) using a surgical blade from five parts (top, bottom, left, right and center) of the same membrane and sputter coated with gold at 10 mA for 20 s before SEM measurement (JEOL JSM-5600LV). All SEM images were taken at a fixed magnification of ×100. Pore size was measured using the software SmileView (Version 2.05).

#### 3.2.4. Surface Roughness Measurement

The surface roughness, R_a_, of the PCL membranes was measured by using the Confocal Imaging Profiler (PLμ, SENSOFAR, Terrassa, Spain). For each size of powder particle, six membranes were investigated with four readings taken at four different positions. 

#### 3.2.5. Mechanical Tests

Mechanical properties of the PCL membranes were tested at room temperature using a Model 5547 Microtester (Instron, Norwood, MA, USA). A total of 10 samples (10 mm × 15 mm) were cut from two PCL membranes for each powder particle size and prepared by sandwiching their two extreme ends between two pieces of cardboard. The cardboard ends were then mounted onto the clamps of the Instron Microtester. Loads of 50 N and strain rate of 10 mm/min were applied.

### 3.3. In Vitro Evaluation of Membranes for Cell Compatibility 

#### 3.3.1. Cell Culture

For cell culture tests, only membranes made from 100 µm PCL powder were used. C2C12 murine myoblasts were plated on the PCL membrane coated wells of a 24-well plate. PCL membranes were held in position with circular hollow metal rings to prevent them from floating. Before cell seeding, PCL membranes were sterilized using 70% ethanol for 1 h and washed with phosphate buffer solution (PBS) several times. Cells were seeded at the density of 2 × 10^5^ cells/well. Cells were cultivated in low glucose Dulbecco’s Modified Eagle’s Medium (DMEM, Sigma, St. Louis, MO, USA) supplemented with 10% FBS (PAA, Pasching, Austria) and 1% antibiotic/antimycotic solution (PAA). Culture medium was replaced after every 2–3 days and cells were grown at 37 °C in the presence of 5% CO_2._ For cell detachment purpose, 0.25% trypsin-EDTA (Invitrogen, Carlsbad, CA, USA) solution was utilized.

#### 3.3.2. Immunocytochemistry and Microscopy

Cells were fixed for 10 min in 4% paraformaldehyde and permeabilized for 15 min in 0.1% Triton X-100 at room temperature. Having washed the cells twice with PBS, for blocking purpose, 5% bovine serum albumin was used for 1 h at room temperature. Then, cells were incubated with primary antibody against mouse monoclonal anti heavy chain cardiac myosin (1:400, Abcam, Cambridge, UK) overnight at 4 °C, followed by washing with PBS for three times. After 1 h incubation with Alexa Fluor 488 goat anti mouse IgG (1:400, Molecular Probes, Eugene, OR, USA), cells were rinsed with PBS several times. Cell nuclei were stained with 4′,6-diamidino-2-phenylindole (DAPI, 1:400, Chemicon, Temecula, CA, USA). To validate the performance of heavy chain cardiac myosin primary antibody, C2C12 myoblasts were cultured in 2% horse serum to induce myogenic differentiation and stained for myosin heavy chain. Images were captured using an Eclipse 80i upright microscope (Nikon, Tokyo, Japan) with X10 objective lens and image analysis was done by using ImageJ 1.44f software.

#### 3.3.3. Cell Proliferation Assay

A PicoGreen assay was implemented to check the proliferation rate of C2C12 myoblasts seeded on the PCL membrane. Initially, the PCL membranes covered with cells were rinsed carefully three times in PBS and immersed in 0.5% Triton X-100 solution to permeabilize cells with gentle pipetting for 30 min. According to the manufacturer’s instructions, PicoGreen working solution was prepared by diluting concentrated PicoGreen reagent in TE buffer (1:200). Then, equal quantities of cell lysate and PicoGreen working solution were mixed together in the wells of a 96-well plate and incubated for 5 min at room temperature. Finally, fluorescence emission readings were taken using an Infinite200^®^ microplate reader (Tecan Inc., Mannendorf, Switzerland) at 520 nm under an excitation wavelength of 480 nm. Cell proliferation rate was measured at different time intervals ranging from day 1 after cell seeding to day 6.

## 4. Conclusions

A novel solvent-free surface suspension method to rapidly fabricate a micro-porous thin membrane scaffold has been developed, which is simple, fast and efficient. The method is distinct from currently known methods such as solvent casting, electrospinning and biaxial drawing, offering an additional option for making micro-porous membrane scaffolds. The process involves three simple steps: preparation of a layer of powder particles to form a surface suspension system, heating and cooling down. The membrane can be fabricated based on the principle of surface tension of hydrophobic particles in two different states. This method eliminates the use of organic solvent and allows formation of an inherently micro-porous membrane within a short period of time. Cell culture experiments with C2C12 mouse myoblast cell showed that the membrane supports cell growth and tissue formation. Thus, it is feasible to be used as a solid scaffold based for muscle tissue engineering. This novel membrane powder-based fabrication technique has proven that it has potential use for TE applications. There are other biodegradable polymers that can be used to replace PCL such as PLLA and PLGA in order to expand the material range, scope and allow more possible applications by using this fabrication method.

## Figures and Tables

**Table 1:** Summary of membrane fabrication processes and morphology.

Method	Processing and Membrane Morphology					
	Fabrication Duration	Organic Solvent Involving	Pore Structure	Texture	Thickness	Reference
Solvent (solution) casting	Hours to Days	Yes	Insufficient pores; Require post-processing	Flat Solid	Depends on concentration	[10]
Biaxial-drawing	Hours	Depends on film preparation	Insufficient pores; Require post-processing	Flat Solid	Ultra-thin	[5,11]
Electrospinning	Hours	Yes	Micro-nano pores	Random fibers structure	Dense ultra-thin	[12,13]

**Table 2:** Membrane characteristics and mechanical properties.

Parameter	Membrane Properties	
	From 100 µm Powder	From 500 µm Powder
Thickness	27.3 ± 2.8 µm	134.9 ± 3.6 µm
Roughness	3.4 ± 2.9 µm	5.5 ± 3.0 µm
Stiffness	2.40 ± 0.40 N/mm	0.15 ± 0.02 N/mm
Ultimate tensile load	1.6 ± 0.3 N	10.1 ± 2.5 N

**Table 3:** Distribution of pore size of three membranes made from 100 µm and 500 µm powder.

Powder Size	Sample	1	2	3
100 µm	Number of pores measured	949	885	1000
	Average (µm)	16.2 ± 9.2	16.7± 10.9	16.2 ± 6.3
	Max size (µm)	95	80	46.1
	Min size (µm)	2	3	6
500 µm	Number of pores measured	996	1016	1000
	Average (µm)	151.7 ± 70.7	61.2 ± 30.5	73.3 ± 26.2
	Max size (µm)	350	288	347
	Min size (µm)	14	11	14
